# Lanternfish (Myctophidae) Zoogeography off Eastern Australia: A Comparison with Physicochemical Biogeography

**DOI:** 10.1371/journal.pone.0080950

**Published:** 2013-12-11

**Authors:** Adrian J. Flynn, N. Justin Marshall

**Affiliations:** 1 The University of Queensland, School of Biomedical Sciences/Queensland Brain Institute, St. Lucia, Queensland, Australia; 2 Commonwealth Scientific and Industrial Research Organisation. Marine and Atmospheric Research, Hobart, Tasmania, Australia; 3 Ichthyology, Museum Victoria, Melbourne, Victoria, Australia; Ecole Normale Supérieure de Lyon, France

## Abstract

In this first attempt to model the distributions of a mesopelagic fish family at this scale in the eastern Australian region (10°S to 57°S), lanternfish species occurrence data spanning a period from 1928 to 2010 were modelled against environmental covariates. This involved: (1) data collation and taxonomic quality checking, (2) classification of trawls into “horizontal” (presence-absence) and “oblique” (presence-only) types, and classification of vertical migration patterns using existing literature and the species occurrence database, (3) binomial GAMs using presence-absence data for representative temperate, subtropical and tropical species to examine depth interactions with environmental covariates and refine the selection of environmental layers for presence-only MAXENT models, (4) Presence-only MAXENT modelling using data from all trawls and the reduced environmental layers, and (5) Multivariate analysis (area-wise and species-wise) of the resulting matrix of logistic score by geographic pixel. We test the hypothesis that major fronts in the region (Tasman Front, Subtropical Convergence, Subantarctic Front) represent zoogeographic boundaries. A four-region zoogeographic scheme is hypothesised: Coral Sea region, Subtropical Lower Water region, Subtropical Convergence/South Tasman region and Subantarctic region. The Tasman Front, Subtropical Convergence and Subantarctic Front represented zoogeographic boundaries. An additional boundary at ∼25°S (coined the ‘Capricorn’ boundary) was adopted to delineate the Coral Sea from Subtropical Lower Water regions. Lanternfish zoogeographic regions are congruent with some aspects of two prevailing physicochemical biogeographic schema in the region, but neither of these schema alone accurately predicts lanternfish distributions. As lanternfishes integrate vertical ocean processes, the hypothesised lanternfish zoogeography may represent a useful model for a generalised pelagic biogeography that should be tested for other oceanic groups.

## Introduction

Lanternfish assemblages are geographically delineated in many ocean regions including the Southern Ocean [1], [2], [3], [4], Canary Islands [5], Humbolt Current region of South America [6], Kuroshio Current region of the northern Pacific Ocean [7], [8], Atlantic Ocean [9], [10], [11], Indo-West Pacific Ocean [12] and California Current region [13]. Temperature (at the surface or at depth) and productivity are cited as important predictors of distributions, although the mechanisms by which environmental parameters influence distributions are often unclear [14], [15], [16]. Because of this link between species distribution and oceanography, lanternfishes [1], [9], [12], [17], [18] and other mesopelagic fishes [14], [19], [20], [21] have been used to derive pelagic biogeographic schema. At the ocean basin scale, pelagic biogeography is generally consistent with present-day or ancestral water mass distributions [22], [23] with fronts functioning as barriers to species distribution or mixing sites [24]. However, existing schema do not suitably resolve regions in waters off eastern-southeastern Australia.

Two prevailing pelagic biogeographic schema have been developed in the eastern-southeastern Australian region that are based on physicochemical properties. The scheme of Longhurst [25], [26] is based on seasonal cycles of mixed layer productivity and consists of four regions in the area. Condie and Dunn [27] erected an alternative scheme based on seasonal characteristics of several parameters of the mixed layer. Condie and Dunn's [27] scheme also resolved four regions in the study area, but differed from that of Longhurst [26]. Condie and Dunn [27] identified a biogeographic boundary at approximately 25°S delineating the Coral Sea from Subtropical Lower Water in the northern Tasman Sea. Longhurst [26] did not recognise a boundary in this area. Longhurst [26] recognised a boundary at the Tasman Front, an eddy-dominated frontal system at interface between the East Australian Current and colder waters of the Subtropical Front to the south. Condie and Dunn [27] did not recognise a biogeographic boundary associated with the Tasman Front.

The vertical nature of lanternfish life-history presents several challenges for species-habitat modelling. Current driven transport processes are likely to affect early life-history stages, which are usually planktonic [28], differently than vertically migrating adults. Among adults, vertical migrators are exposed to transport processes that non-migrating species are not. Further, vertical migration behaviours expose lanternfishes to a wide range of environmental conditions throughout a single day and there are likely to be complex interactions between pressure, light, metabolism, energetics and feeding that combine to create preferential 3-dimensional habitats and place limits on distributions. Indeed, distributions can change where vertical niches, that are specific to life-history stages, intersect with seabed topography or isolated oceanographic features [29], [30], [31]. These factors complicate any simplistic view of the mechanism whereby environmental tolerances control lanternfish distributions. Finally, dependable species-area analyses in 3-dimensional mesopelagic habitats require data that are replicated on horizontal and vertical planes. Such replicated depth-stratified sampling is not available for the whole area considered in the present study.

This study addressed these problems in three ways. First, by using data from collections spanning some 80 years, the analysis of species distribution incorporated juvenile and adult distributions over a range of seasonal and decadal cycles. Therefore, derived distributions should represent species ranges that have become stable over the long-term and that integrate vertical migration behaviour. Second, a blend of presence-absence and presence-only modelling techniques were used to examine species-habitat relationships and refine the selection of environmental covariates included in models, restricting the potential for model over-fitting.

Phytoplankton distributions [32], [33], [34] and biochemical signatures [35], [36] have been incorporated into some bioregionalisation analyses in the eastern Australian region. However, to date no attempts have been made to investigate distributions in a family of oceanic pelagic fishes and how these map onto existing physicochemical schema. This study aimed to investigate relationships between depth-stratified environmental predictors and the distribution of lanternfish species and to investigate the latitudinal distribution of species groups. We aimed to test the hypothesis that lanternfish assemblages in oceanic waters off eastern and southeast Australia are demarcated by major oceanographic frontal systems; namely the Tasman Front (TF), Subtropical Convergence (STC) and Subantarctic Front (SAF). We erect a hypothesised lanternfish zoogeography for the region, compare this against two prevailing physicochemical schema and discuss the utility of this family of fishes as a model for pelagic biogeography.

## Methods

### Data Collation

Lanternfish species records spanned the years 1928 to 2010 and were collated from five sources: (1) Museum collection data: Australian Museum, National Museum of Victoria, Queensland Museum, Museum of Comparative Zoology (Harvard University), Natural History Museum of Denmark (Copenhagen); (2) Taxonomic collection data from research institutions: CSIRO Marine and Atmospheric Research (CSIRO) Australian National Fish Collection, Australian Antarctic Division Fish Collection; (3) Lanternfish catch composition data from published papers and fisheries reports; (4) Unpublished lanternfish catch composition data from fisheries (CSIRO) and biodiversity (Australian Institute of Marine Science) surveys; (5) New, previously unpublished, collections made in the Coral Sea [37] and the Tasman Sea abyssal basin [38].

### Taxonomy and distribution quality control

Taxonomic data quality control involved checking species records against known species in Australian waters. The primary reference for this was Paxton and Williams (unpublished data) but included other family-wide taxonomic resources as such [2], [39], [40], [41], [42], [43], [44], [45]. Non-recognised or non-valid names were checked against updated classifications made since the original identification. The primary reference for this was the California Academy of Sciences online Catalog of Fishes database [46]. Records from Australian collections that contained non-valid species names that were unable to be validated in the steps above were re-identified by examining museum-held specimens.

In cases where the identification of damaged or small juvenile specimens was not able to be resolved, spurious records were deleted. Records from overseas collections containing species names that were unable to be validated were deleted. All species records were mapped. Where there was a large geographic gap in the northern and southern extents of the distribution records for a species, identification checks were made on Australian museum-held specimens from the northern-most and southern-most areas. Similarly, if a species was distributed continuously over a very large latitude range, identification checks were made on representative specimens from the northern-most and southern-most limits of the distribution.

Two additional quality control rules were imposed. Species that were represented by a single record and species that were represented only in a single 1-degree latitudinal band were deleted. These two final quality control steps resulted in the deletion of 10 species from the analyses: *Bolinichthys distofax, Centrobranchus andreae, Diaphus adenomus, Diaphus chrysorynchus, Diaphus coeruleus, Diaphus whitleyi, Loweina interrupta, Loweina rara, Myctophum lunatum, Protomyctophum luciferum.*

A depth cut-off of 1000 m was applied and any species records beyond this depth were omitted from analysis.

### Study boundaries and species records

**Figure 1:**
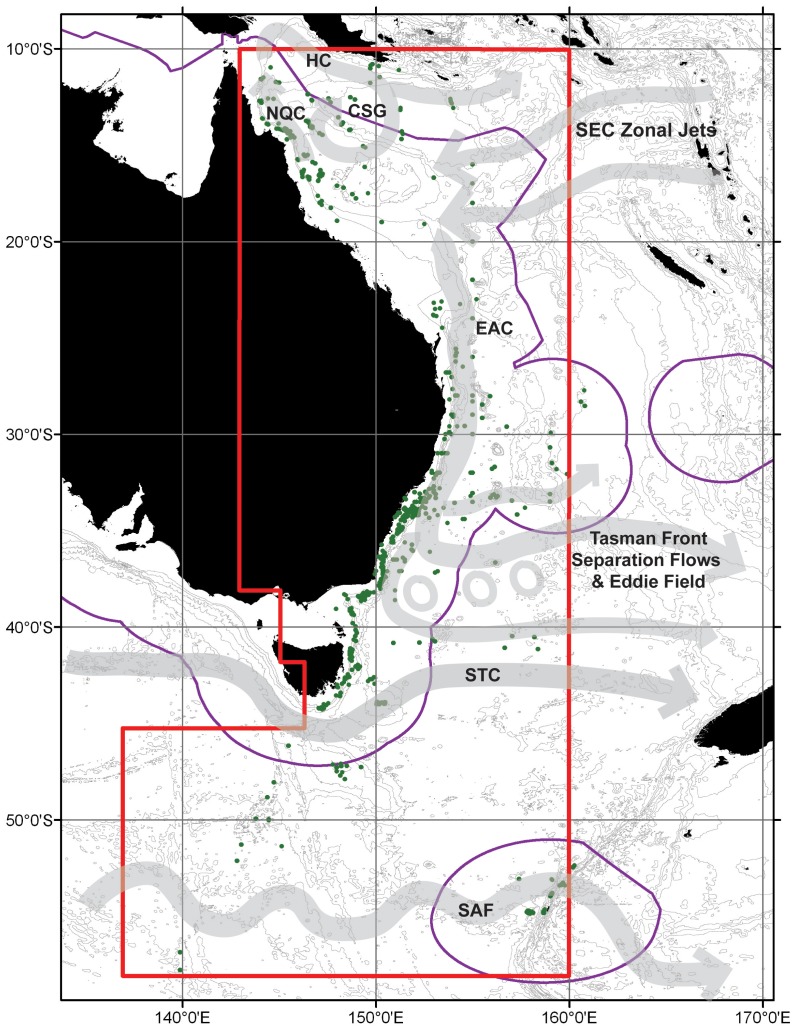
Study boundary (red line) and location of lanternfish species occurrence records (green dots). Major currents and frontal systems shown schematically by grey arrows (SEC = South Equatorial Current, NQC = North Queensland Current, HC = Hiri Current, CSG = Coral Sea Gyre, EAC = East Australian Current, STC = Subtropical Convergence, SAF = Subantarctic Front). Purple line indicates the Australian Exclusive Economic Zone (EEZ). 200 m bathymetric contour denoting the continental shelf and subsequent 1000 m intervals shown.

The geographic boundaries of the study, the location of species records and major currents and frontal systems in the region are shown in Figure 1. The latitudinal extent of the study area was 10°S to 57°S inclusive, corresponding to tropical waters of the Coral Sea to Subantarctic waters of the Southern Ocean. The eastern boundary was 160°E and the western boundary of the study was latitude dependent. The western part of Bass Strait and western Tasmania were excluded as they are regularly bathed by the Zeehan Current (an extension of Lueewin Current) [47] and thus potentially include expatriate species from southern and western Australia. In the Southern Ocean, the western boundary was set at 136°E so as to include the maximum number species records in this region. Lanternfish species in the Southern Ocean are generally distributed throughout a wide longitudinal range [2] and so there was low risk of including non-representative species by extending the study boundary to the west in this way.

### Trawl types and depth records

Lanternfish species records were derived from horizontal trawls and oblique trawls. Horizontal trawls used nets (often closing-nets or closing cod-ends) that were trawled through depth strata less than 200 m wide. The depth ascribed to a species record from a horizontal trawl was the mid-point of the depth trawled. Records from horizontal trawls were treated as presence-absence data.

Oblique trawls used non-closing nets that trawled through a depth range greater than 200 m and the majority of museum records were of this type. Records from oblique trawls were treated as presence-only records. A method was developed to ascribe a depth to species records from oblique trawls. First, the vertical migration behaviour of each species was coded (Table 1) based on literature review and data compiled for the present study. Second, the maximum and minimum depth range of species was collated from existing literature and observations from the horizontal trawl data from the present study. Third, a single species record from an oblique trawl was expanded to ascribe a minimum, maximum and centroid depths to a record, with a minimum depth cut-off of 10 m and a maximum depth cut-off of 1000 m. For example, in an oblique trawl from 0 to 850 m, a record of a species with broad-ranging vertical migration (e.g. migrator code = 1) was expanded to 3 records with depths ascribed as 850 m, 425 m, and 10 m.

**Table 1:** Codes assigned to describe the vertical migration characteristics of lanternfish species.

Migrator Code	Description
1	Large range nyctoepipelagic migration from at least 1000 m to 0–100 m
2	Moderate range nyctoepipelagic migration from 500–1000 m to 0–100 m
3	Small range nyctoepipelagic migration from less than 500 m to 0–100 m
4	Large range lower mesopelagic to upper mesopelagic migration from >1000 m to 100–200 m, not reaching epipelagic zone
5	Moderate range lower mesopelagic to upper mesopelagic migration from >1000 m to 200–500 m
6	Small range lower mesopelagic to upper mesopelagic migration from 500–1000 m to 100–400 m
7	Upper mesopelagic non-migrator (200–500 m)
8	Lower mesopelagic non-migrator (500–1000 m)
9	Bathypelagic non-migrator (>1000 m)

The total number of species_×_location_×depth_ records was 4,912. 2,311 (47%) records were derived from oblique trawls and 2,601 (53%) records were derived from horizontal trawls.

### Data analysis

A two-stage approach was used to handle the two types of data in this study: presence-absence data from horizontal trawls and presence-only data from oblique trawls. First, GAMs were used to explore species-habitat relationships for three species that represented a tropical, subtropical and temperate species and that were present in large numbers in horizontal trawls (presence-absence data). GAMs were used to examine interactions between depth and environmental predictors. The results of GAMs were used to refine the selection of environmental predictors that were later used in presence-only models (MAXENT) for all species over the full study area, that required the use of presence records from both oblique and horizontal trawls.

#### Generalized Additive Models

The 200 m bathymetry contour (nominated as the continental shelf break) formed the western limit of the geographic extent of predictions generated from Generalized Additive Models (GAMs).

GAM [48] is a generalized linear model where the linear predictor is specified as the sum of smooth functions of some or all of the explanatory variables [49]. A GAM takes the form:
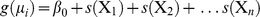


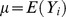
Where *Y_i_* ∼ an exponential family distribution, *g* is a monotonic link function, *β* is a fixed parameter and *s* is a smoothing function of covariates X*_i_*.

The method is an effective exploratory tool for presence-absence data (binomial distributions) [50], [51] and has been used to model pelagic fish species habitats [16], [52], [53], [54].

Environmental covariates were obtained from the CSIRO Atlas of Regional Seas (CARS) 2009 at a resolution of 0.5° grid. Temperature, salinity, phosphate, nitrate and oxygen were modelled to the matching *x*,*y*,*z* locations of species records [55]. For these five parameters, mean values and seasonal amplitude were available for use in models. Latitude, bottom depth, mixed layer depth, surface primary production and chlorophyll-a concentration were available at the matching species *x*,*y* locations. Salinity: temperature ratio was calculated from mean values of temperature and salinity for use in GAMs.

Models were constructed in R (R Development Core Team 2011) using the mgcv library [49], [56]. A binomial error distribution and logit link function was used, and the natural cubic spline smoother was adopted after confirming that different smoothing bases had no significant effect on models. Models were developed for three species: *Electrona risso* (temperate), *Diaphus mollis* (subtopical) and *Diaphus luetkeni* (tropical). Models were constructed using a stepwise forward selection method in order to obtain the simplest model and avoid collinearity. Interactions between explanatory variables and depth were examined before dropping interaction terms from models. Models were compared by assessing the Akaike Information Criterion (AIC) scores [57] and the anova.gam function in the ‘mgcv’ library. The final GAM model was used in the predict.gam function of the mgcv library to predict species distribution onto a background 0.5° grid of the study area. The model prediction onto the background 0.5° grid was performed at three depths: 0 m, 150 m and 500 m.

The modelled species predictions were rasterized in ArcMap (ESRI, Redlands, CA, USA) and mapped along with all actual species occurrence records as another check of performance. The standard error of model predictions was also mapped to visualise areas of uncertainty.

#### MAXENT models

After refining the selection of environmental variables based on the GAM results, presence data for all species recorded from oblique and horizontal trawls were included in maximum entropy modelling (MAXENT v3.3.3a), a machine-learning technique that has been shown to perform well with presence-only data [58]. MAXENT was set to automatically select feature types from a possibility of linear, quadratic, product, threshold or discrete relationships applied between environmental layers and the species layer to constrain the probability distribution of species occurrence. The environmental variables used in presence-only models were: latitude; bottom depth; mean values for temperature, salinity, phosphate, nitrate and oxygen at 0 m, 150 m and 500 m depth strata.

The MAXENT regularisation multiplier was set to the default level of 1 to avoid over-prediction beyond the geographic range of records. The species presence records were divided into 75% training and 25% test data sets. Resulting models for each species were validated using the area under the receiver operating characteristic curve (AUC) and binomial tests. Species with a test AUC of <0.75 were excluded from further analysis following O'Hara et al. [59]. Using this AUC cut-off criterion, an additional 18 species were excluded from the final analysis based on MAXENT logistic scores: *Bolinichthys photothorax*, *Bolinichthys prysobolus*, *Centrobranchus nigroocellatus*, *Diaphus anderseni*, *Diaphus fulgens*, *Diaphus problematicus*, *Electrona carlsbergii*, *Gymnoscopelus hintinoides*, *Gymnoscopelus opisthopterus*, *Lampadena anomala*, *Lampadena speculigera*, *Lampadena urophaos*, *Metelectrona herwigi*, *Myctophum brachygnathum*, *Myctophum obtusirostre*, *Protomyctophum choriodon*, *Symbolophorus boops* and *Taaningichthys bathyphilis*.

The background logistic scores for each species-habitat model were trimmed to the geographic boundaries of the study. The resulting data matrix contained logistic scores for 95 species at 2589 geographic pixels. The logistic output matrix was used directly in multivariate analysis.

#### Area and species multivariate analyses

The matrix of species logistic scores by geographic pixel, derived from MAXENT, was analysed using a quantitative form of the Ochiai similarity measure [60] to create area-wise and species-wise resemblance matrices. The Ochiai similarity measure is in the Bray-Curtis family of measures, the behaviour of which is intermediate between the Bray-Curtis and Kulczynski measures [61], and maintains the independence of joint absence criteria (i.e., taxa which are not present in either sample do not affect the resemblance between two samples). Multivariate analyses were performed using the PRIMER v6 software package [60]. Area-based multivariate analysis was performed and samples were coded by latitude and the resulting clusters were labelled based on their location in relation to the following water masses and major oceanographic fronts: (1) 10–24°S, Coral Sea [27], [62]; (2) 25–35°S, Subtropical Lower Water (STLW) [27], [47]; (3) 32–35°S, Tasman Front (TF) [63], [64]; (4) 44–49°S, Subtropical Convergence (STC) [65], [66], [67]; (5) 50–54°S, Subantarctic Front (SAF) [68], [69], [70]; (6) 55°S+, Subantarctic below SAF.

The area-based analysis resulted in a five-cluster dendrogram that grouped geographic pixels in relation to their location with respect to the six *a-priori* water masses. Species-based multivariate analysis was then conducted and species were coded by their recorded occurrence in each of the five water mass clusters generated from the area-based analysis. The species-based analysis identified a four-cluster solution and this was selected in preference to the area-based analysis to represent a lanternfish zoogeographic hypothesis as it gave priority to observed species ranges.

The species-area dendrogram was coded according to the actual recorded species presence over their entire latitudinal range. In the absence of standardised abundance data that could be used to quantitatively assess “core” and “expatriate” distributions, this qualitative method explicitly identifies the full geographic range of species forming clusters.

To visualise the identified cluster groups with respect to geographic location, the mean logistic score per geographic pixel was calculated by summing the logistic scores for each species comprising a cluster and dividing by the total logistic scores for all species. This mean logistic score per geographic pixel (0.5°) was rasterized in ArcMap (ESRI, Redlands, CA, USA) and re-processed to 0.05° resolution using inverse distance weighted interpolation of logistic scores at fixed search radius = 1.

## Results

### Generalized Additive Models

#### Electrona risso – Temperate species

The model with the best performance for *E. risso* took the form:
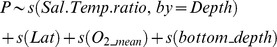
Where *P* = presence of *E. risso*, *Sal.Temp.ratio* = salinity: temperature ratio, *Depth* = 3-category depth factor of applied to model predictor variables (0–50 m, 51–150 m, 151–500 m), *Lat* = latitude, *O_2__mean* = mean oxygen concentration, *bottom_depth* = depth from the surface to the seafloor.

**Figure 2:**
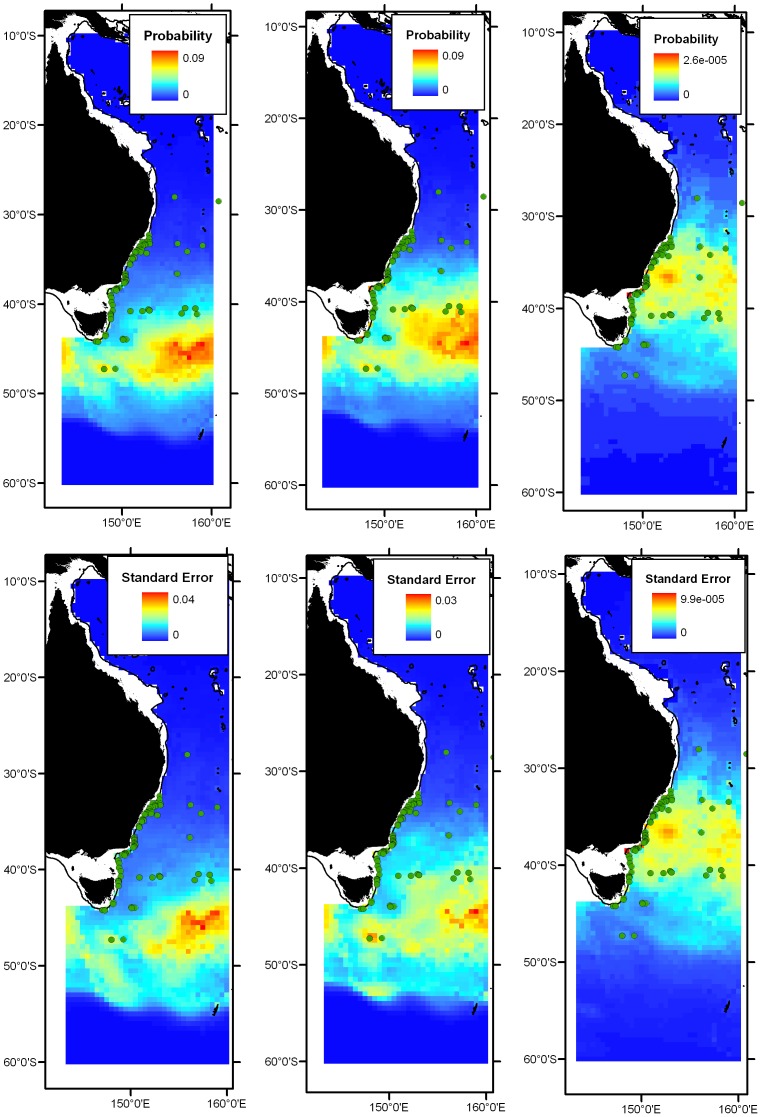
Predicted distributions of *Electrona risso* (top row) and standard errors (bottom row). From left to right: 0 m, 150 m and 500 m depth envelopes. Green dots indicate all occurrence records of *E. risso*.

Model predictions at the 0 m, 150 m and 500 m depth envelopes are shown in Figure 2. In surface waters, the modelled distribution of *Electrona risso* is centred in the STC at the boundary of the Tasman Sea and Southern Ocean. At the 150 m depth envelope, the modelled distribution shifted to the north, and improved the prediction of occurrences on the eastern Tasmanian and Victorian continental slopes. At the 500 m depth envelope, predicted distributions spread further to the north, improving the prediction of occurrence records from the NSW continental slope and the northern-most records for the species. The predicted distributions of *E. risso* may reflect the northward penetration of cold STC water in deep layers off southeastern Australia lying underneath warmer subtropical surface waters.

#### Diaphus mollis – Subtropical species

Data for this species were sufficient to model distributions at 0 m and 150 m. The additive model with the best performance for *D. mollis* took the form:
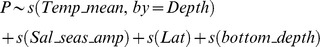
Where *P* = presence of *D. mollis*, *Temp_mean* = mean temperature, *Depth* = 3-category depth factor of applied to model predictor variables (0–50 m, 51–150 m, 151–500 m), *Sal_seas_amp* = seasonal amplitude of salinity, *Lat* = latitude, *bottom_depth* = depth from the surface to the seafloor.

**Figure 3:**
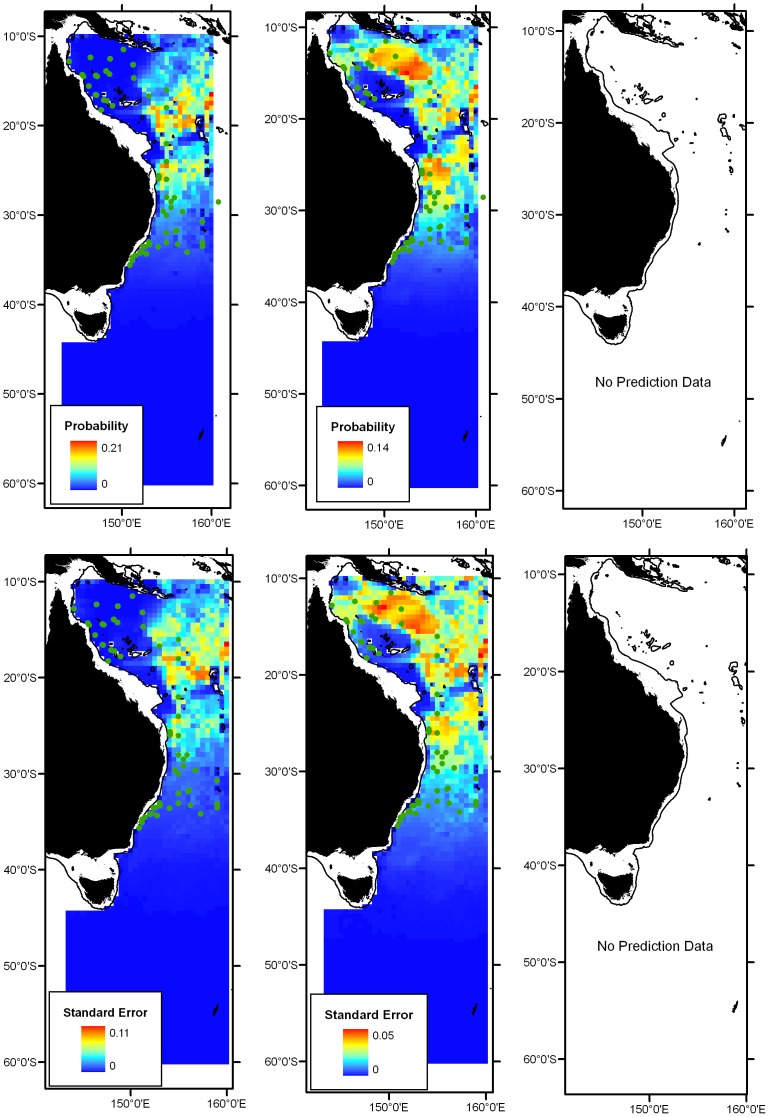
Predicted distributions of *Diaphus mollis* (top row) and standard errors (bottom row). From left to right: 0 m, 150 m and 500 m depth envelopes. Green dots indicate all occurrence records of *D. mollis*.

The model predicts a southern limit to the distribution of *D. mollis* corresponding with the Tasman Front (Figure 3). The model for the 150 m depth envelope extended the predicted distribution of *D. mollis* into the northeastern sector of the Coral Sea to encompass occurrence records in this area that was not predicted in the 0 m envelope.

#### Diaphus luetkeni – Tropical species

The model for *D. luetkeni* took the form:
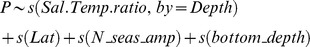
Where *P* = presence of *D. luetkeni*, *Sal.Temp.ratio* = salinity: temperature ratio, *Depth* = 3-category depth factor of applied to model predictor variables (0–50 m, 51–150 m, 151–500 m), *Lat* = latitude, *N_seas_amp* = seasonal amplitude in nitrate concentration, *bottom_depth* = depth from surface to the seafloor.

**Figure 4:**
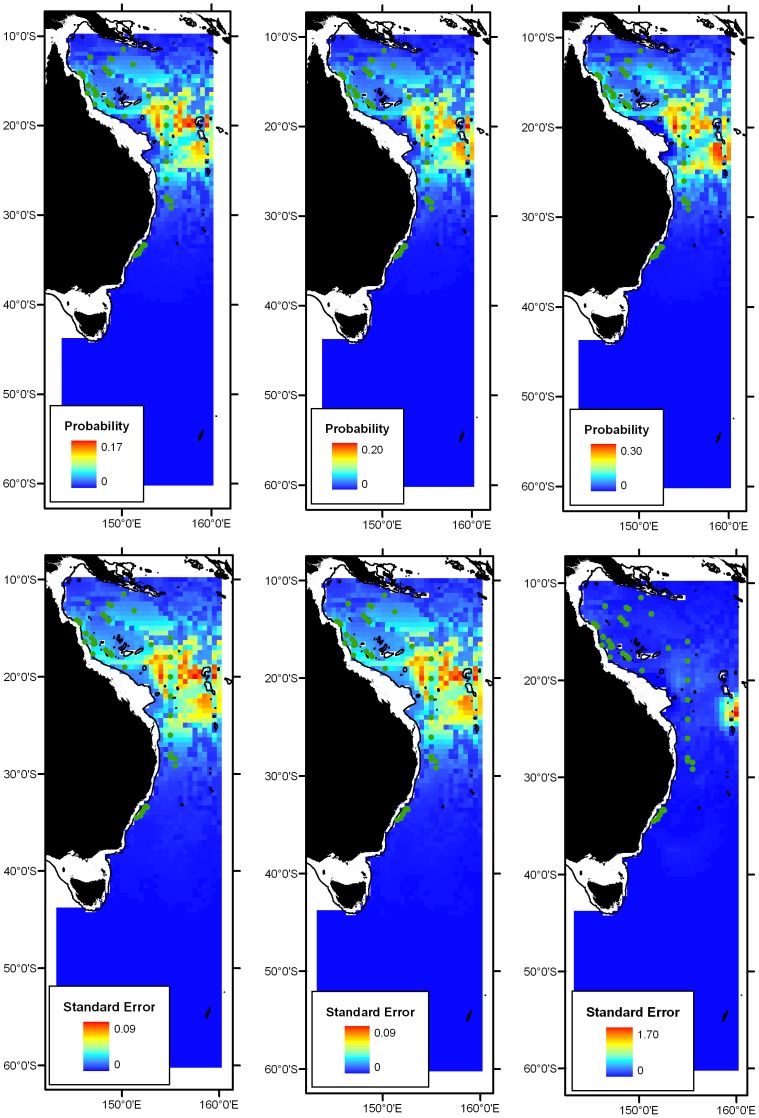
Predicted distributions of for *Diaphus luetkeni* (top row) and standard errors (bottom row). From left to right: 0 m, 150 m and 500 m depth envelopes. Green dots indicate all occurrence records of *D. luetkeni*.

Models at the 0 m, 150 m and 500 m depth envelopes predict the distribution of *D. luetkeni* in the Coral Sea, with a southern limit at approximately 25°S (Figure 4). The ability for models to predict occurrences of the species in the far northwestern region of the Coral Sea off Cape York was relatively poor. Occurrences of *D. luetkeni* off central NSW were not predicted by the model.

#### Key GAM results

For the three species tested, mean temperature or salinity: temperature ratio was the primary model terms and the performance of all models was improved significantly by introducing a three category depth factor (0–50 m, 51–150 m, 151–500 m) to these terms. Therefore, the environmental layers used in MAXENT presence-only models were selected at those three depth-envelopes.

The performance of all models was improved by inclusion of latitude and bottom depth. These two terms were therefore included in MAXENT models. Chlorophyll-a and primary production did not significantly improve performance in the any of the GAMs presented and thus were excluded from MAXENT models.

### MAXENT Models

The area-based dendrogram of logistic score×geographic pixel, derived from MAXENT species-habitat modelling, is given in Figure S1. A five-cluster area-based hypothesis was identifiable at ∼52% similarity level. At a low similarity level (20%), sites located north of the Tasman Front (TF) were separated from those located south of the TF, indicating the importance of this frontal system as a zoogeographic boundary. North of the TF, sites in the Coral Sea were clustered separately from STLW locations. South of the TF, SAF/Subantarctic sites grouped together and sites in the STC were separated from those in the southern Tasman Sea.

**Figure 5:**
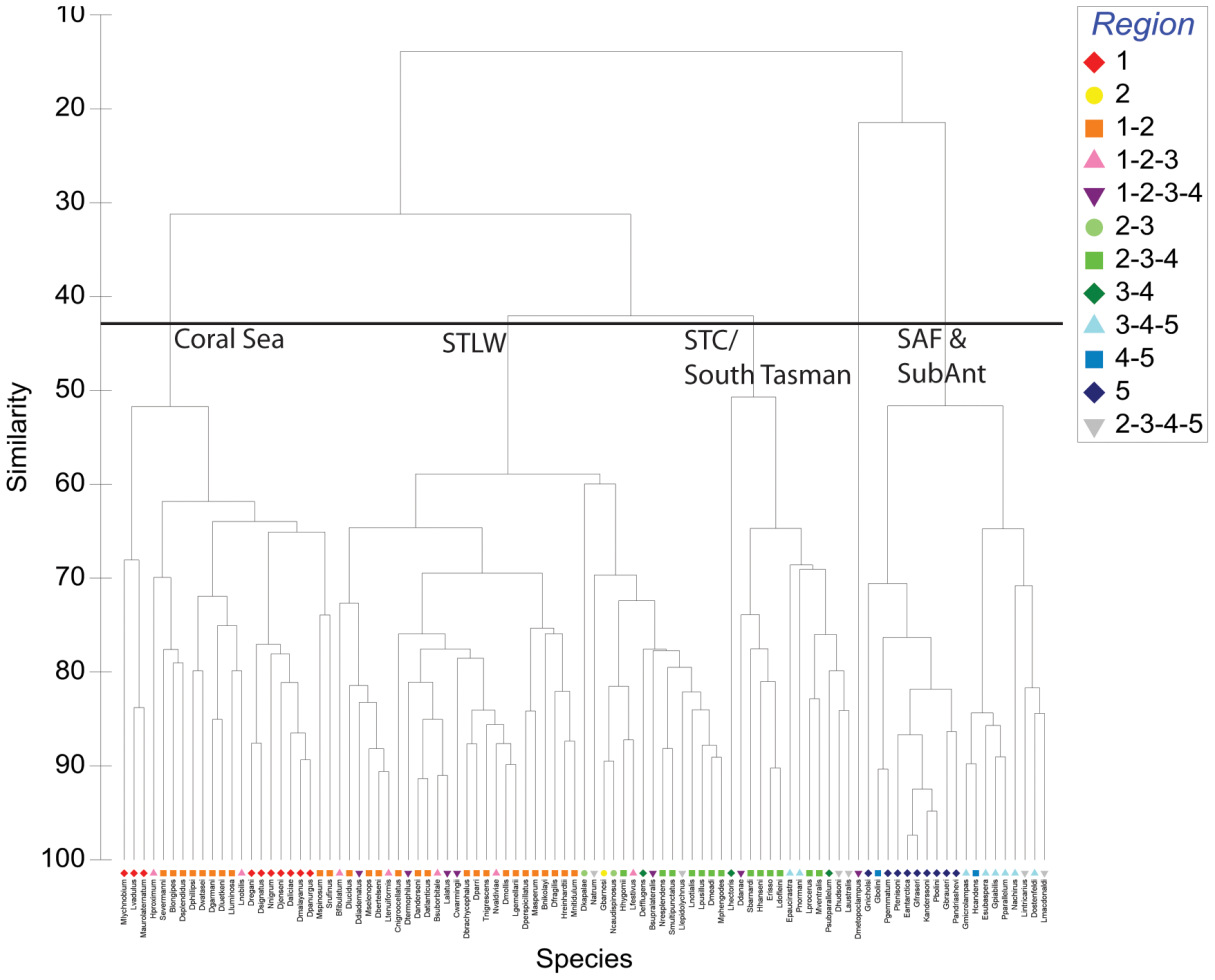
Species-wise dendrogram of MAXENT logistic scores (Ochiai similarity). Species colour-coded by presence within each of 5 regions identified in the area-wise analysis (see Figure S1).

The species-based analysis of the logistic score×geographic pixel matrix identified a 4-cluster hypothesis at the 45% similarity level (Figure 5). Species-based analysis identified clusters that were parsimonious with the area-based analysis, with clusters comprising Coral Sea, STLW and SAF/Subantarctic groups. However, the species-based analysis combined the STC with the southern Tasman Sea, creating a single STC/South Tasman group. This 4-cluster species-based hypothesis of classification was selected in to represent a hypothesised lanternfish zoogeographic scheme.

### Contribution of covariates to MAXENT models

**Figure 6:**
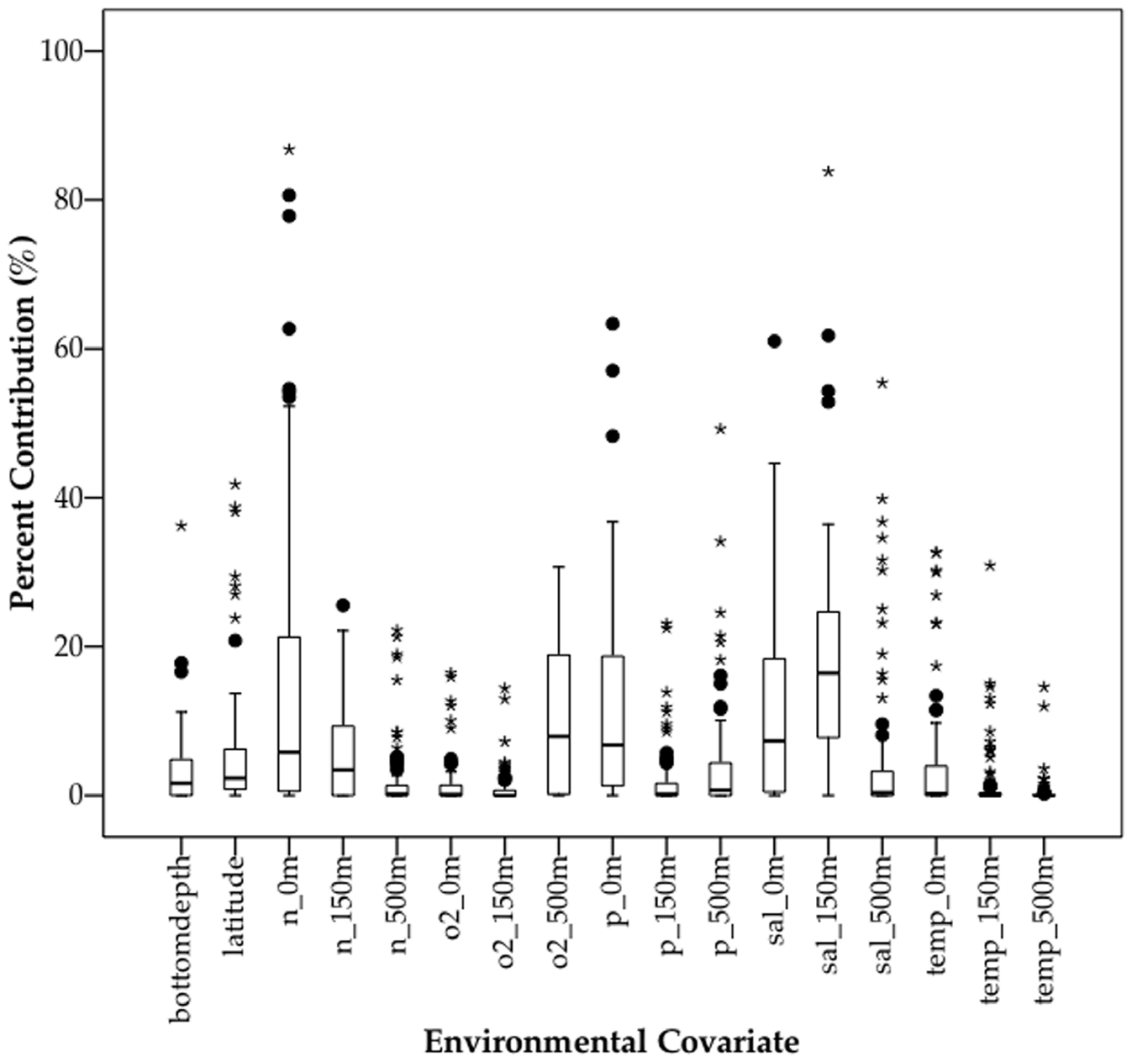
Boxplot summary of percentage contribution of environmental covariates to MAXENT species-habitat models for 95 lanternfish species. Three depth strata (0 m, 150 m and 500 m) tested for variables nitrate (n), phosphate (P), oxygen (O_2_), salinity (sal) and temperature (temp). Boxes represent median and interquartile range, whiskers span data range, black circles represent outliers and stars represent extreme values.

Temperature at 0 m contributed highly to some species models, as did latitude and bottom depth (Figure 6). Nitrate at 0 m and 150 m, phosphate at 0 m, salinity at 0 m and 150 m and oxygen at 500 m were important for some models. The percentage contributions to the MAXENT model should be viewed with caution as they are heuristically defined (i.e., dependent on the particular path that the MAXENT code used to get to the optimal solution) and there are potentially correlated predictor variables [71].

### Zoogeographic regions

**Figure 7:**
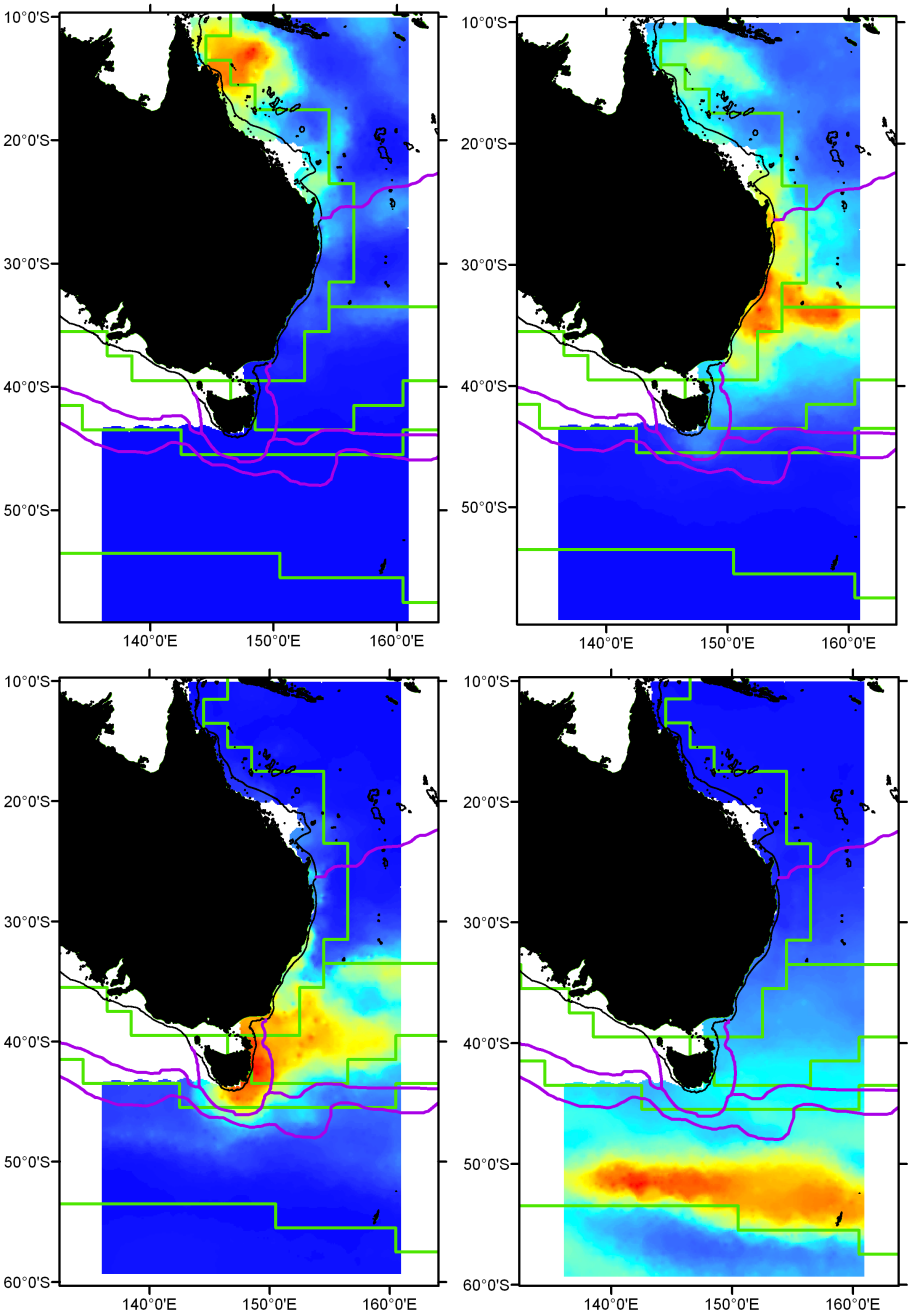
Visualisation of the 4-group hypothesis of lanternfish zoogeographic regions overlaid on biogeographic regions of Longhurst [26] (Green lines) and Condie and Dunn [27] (Purple lines). The heat maps represent mean logistic scores that can be interpreted as model predictions of likelihood of occurrence from high (warm colours) to low (cool colours). Heat maps are provided for each of the 4 hypothesised regions, clockwise from top left: Coral Sea region, STLW region, Subantarctic region, STC/South Tasman region. 200 m bathymetric contour denoting the continental shelf is shown.

The species-wise 4-cluster zoogeographic hypothesis is shown in Figure 7. The biogeographic schemas of Condie and Dunn [27] and Longhurst [26] are also shown in Figure 7. The four nominated lanternfish zoogeographic regions are described as follows: (1) Coral Sea region: distributions from 10°S to approximately 25°S, with a southern boundary conforming to the Coral Sea-STLW boundary of Condie and Dunn [27], coined herein the ‘Capricorn’ boundary, (2) STLW region: distributions extending from Coral Sea to the Tasman Front, with a southern boundary conforms loosely to the northern ‘Tasman Sea’ boundary of Longhurst [26], (3) STC/South Tasman region: distributions centred south of the Tasman Front and spanning the ‘Tasman Sea’ and ‘STC’ regions of Longhurst [26], with the southern limit conforming to southern boundaries of STC zones of Longhurst [26] and Condie and Dunn [27], (4) Subantarctic region: disperse distribution, centred in the SAF but extending north into STC zone. Conforms approximately with ‘Subantarctic’ zones of Longhurst [26] and Condie and Dunn [27].

### Characterisation of zoogeographic regions

The Coral Sea region comprised 22 species, 10 of which had distributions restricted to tropical waters and did not extend south beyond the Capricorn boundary (see Table S1). The STLW region comprised 40 species, including 17 subtropical species that occur in the Coral Sea, but whose southern distributions extended beyond the Capricorn boundary and extend south the Tasman Front (33–35°S). The STLW region also consisted of 10 temperate species with distributions centred in the vicinity of the Tasman Front but also crossed the front and extended further south into higher latitudes of the Tasman Sea, with most not extending as far as the STC boundary (see Table S1).

The STC/South Tasman region comprised 13 species with distributions centred in the southern Tasman Sea with a strong northern boundary at the Tasman Front. Southern boundaries of most high-fidelity species of this region extended south to the southern boundary of STC boundary (see Table S1).

The Subantarctic region diverged from other groups at a low level of similarity and comprised 19 species. Nine species had distributions confined to the SAF (50–54°S) and higher latitudes (>55°S). An additional 10 species had core distributions in the SAF but with ranges that extended north of the SAF into the Southern Ocean (see Table S1).

## Discussion

### Hypothesised Lanternfish Zoogeography

Region-scale structuring of lanternfish species was identified which corresponded to latitudinally-demarcated water masses off eastern and southeastern Australia. We pose a four-region lanternfish zoogeographic hypothesis that is congruent with some aspects of the two prevailing physicochemically-derived schema [26], [27]. However, individually, neither of these two physicochemical schema suitably predicted lanternfish distributions. The four hypothesized zoogeographic regions, from north to south are: Coral Sea region, Subtropical Lower Water (STLW) region, STC/South Tasman region and Subantarctic region.

The major frontal systems of the Tasman Front (TF) and Subtropical Convergence (STC) and the Subantarctic Front (SAF) represented zoogeographic boundaries. The TF has been identified as a mixing zone of warm-water and cold-water species [72], [73], [74] attributable to the complex oceanographic mixing processes of this front [63], [75] and variability in its latitudinal extent [76]. For lanternfishes, the TF appears to be an asymmetrical boundary, representing a semi-permeable southern boundary for the STLW region and a stronger northern boundary for the STC/South Tasman region.

The core of the Coral Sea zoogeographic region was associated with the North Queensland Current (NQC) and the Coral Sea Gyre (CSG), a variable but quasi-stationary clockwise gyre system [77]. The NQC [78] arises from the northern branch of the bifurcation of the South Equatorial Current (SEC) as zonal jets collide with the continental slope at approximately 15–18°S [77]. The potential for the CSG to establish pelagic retention/recirculation currents was identified by Dennis et al. [79], who postulated that the gyre is involved in transport of lobster larvae from the Gulf of Papua to the northern Great Barrier Reef.

Condie and Dunn's [27] physicochemical water mass boundary delimiting the Coral Sea region from the STLW region at ∼25°S (coined the Capricorn boundary herein) was adopted in the present study to represent a hypothesised lanternfish zoogeographic boundary. The Capricorn boundary corresponds to a zone of formation and intensification of the East Australian Current that is characterised by complex flow patterns and eddies [64], [80]. Young et al. [81] identified characteristics of the phytoplankton community and primary production environment of EAC-formation and intensification region (∼26°S) that differed from more southern (∼29°S) offshore sites bathed by colder Tasman Sea water. A biogeographic boundary in the Capricorn region at ∼25°S has been identified for a range of marine groups including shallow water molluscs [82], fishes [83] and intertidal organisms [84].

The Capricorn boundary corresponds approximately with Last et al.'s [85] Central Eastern Transition zone based on demersal fishes of the continental outer shelf and slope. The 25°S area also appears to be congruent with a regional subdivision in sponge fauna [86]. Revill et al. [35] demonstrated a shift in isotopic signatures in pelagic top-predators off the east coast of Australia at latitude of approximately 28°S, attributed by those authors to a water mass discontinuity. Further, Hobday et al. [36] identified that Coral Sea and Western Pacific habitats were demarcated from Tasman Sea and more southerly habitats at mean latitude of approximately 27°S. However, biogeographic schema based on primary productivity [26] and chlorophyll-a [87] did not predict the occurrence of a boundary in this area.

The core of the hypothesised STLW zoogeographic region approximates Briggs' [88] Southeastern Australian Province (retained by Briggs and Bowen [89]) and Last et al.'s [85] Central Eastern Province. The STC/South Tasman lanternfish region corresponds approximately to the latitudes of Last et al.'s [85] Tasmanian province. The observed lanternfish zoogeographic boundary at the TF is congruent with Last et al.'s [85] transitional zone (ecotone) between the Tasmanian and Central East provinces. The southern boundary of the STC/South Tasman region corresponds to the southern extent of the STC boundaries of Longhurst [26] and Condie and Dunn [27]. Robertson and Roberts [90] and McGinnis [2] also identified the STC as a zoogeographic boundary. The STC is associated with strong gradients of physical properties in the upper 400 m [66] but is also highly variable [67]. Barange et al. [91] showed that the STC is associated with high biological productivity and can represent a strong boundary in some areas and a weak boundary in others. Pakhomov et al. [92] identified distinct micronekton and zooplankton faunas in the STC and SAF (termed the Antarctic Polar Front by those authors) off Southern Africa.

The Subantarctic region was characterised by core distributions associated with the SAF, a possible artefact of the distribution of samples used in the analysis. Modelled distributions in the Subantarctic region extended north to overlap somewhat with the STC/South Tasman region. The STC appeared to be a stronger boundary for the southerly extent of subtropical species, in agreement with Koubbi et al.'s [1] ecoregionalisation in the Indian Ocean sector of the Southern Ocean. Subantarctic Mode Water penetrates northward from the Southern Ocean underneath the surface waters of the Subtropical Convergence and Tasman Sea [47], [93], [94], [95]. Species that have affinities with Subantarctic water masses may have ranges extending north into the Subtropical Convergence and southern Tasman Sea if they occupy deep strata. Koubbi et al.'s [1] ecoregionalisation using generalised dissimilarity modelling (GDM), indicated that there could be up to three pelagic “ecoregions” between the STC and SAF, although the authors acknowledged that there were relatively few samples in this band.

To successfully predict or monitor biological distributions using oceanographic and other remotely-sensed variables, parity between pelagic biogeochemical provinces and pelagic biological patterns needs to be established [96]. As lanternfish life-history integrates horizontal and vertical processes, zoogeography in this this family may represent a useful model for pelagic biogeography more broadly. Our hypothesis should be tested with further sampling and using other oceanic pelagic families, particularly in the Coral Sea, the Capricorn boundary area, and in the Pacific sector of the Southern Ocean.

### Biological basis for zoogeography

Boltovsky [97] highlighted the conundrum that indicator species initially selected for their fidelity to temperature-salinity ranges are used for defining biogeographic areas and thus biogeographers often inadvertently model oceanography. Further, the present study integrated all life-history stages and temporal cycles that had the potential to mask boundary-forming effects of frontal systems. Despite these problems, the hypothesised lanternfish zoogeography is generally supported by other regionalisation studies [35], [36], [85], suggesting that models resulted in an informative view of large-scale lanternfish distributions.

Distributions of mesopelagic species have been linked to water column parameters, most notably temperature-salinity regimes [9], [19], [98]. Hulley [99] showed that temperature at 200 m depth in Southern Ocean was a useful predictor for lanternfish (tribe Electronini) distribution. Also in the Southern Ocean, Koubbi et al. [1] reported that temperature at 200 m depth contributed more to a model of lanternfish assemblage distribution than sea surface temperature.

In addition to temperature and salinity variables, oxygen and nitrate concentration were important in models for some species in the present study. Hulley and Duhamel [45] indicated that, in addition to thermohaline characteristics, the distribution of some *Bolinichthys* species could be related to oxygen concentrations at 100 and 200 m depth. While the distribution of lanternfishes and other deep-sea species has been related to zones of major oxygen deficiencies [100], biological responses to environmental gradients in the deep-sea pelagic zone are poorly understood.

The inclusion of the term ‘latitude’ significantly improved the performance of models, suggesting that there are environmental gradients that geographically co-vary with lanternfish distributions but are not explained by the parameters examined in this study. Patterns of mesopelagic fish distribution and diversity have been correlated to primary productivity regimes [14], [23]. In the Atlantic Ocean, Fock [101] identified a negative relationship between deep-sea pelagic ichthyoplankton diversity and surface primary production and chlorophyll concentration. In the Southern Ocean, the distribution of *Electrona antarctica* was correlated with low concentrations of chlorophyll-a [16]. In the eastern-southeastern Australian region, gradients of productivity occur and there are seasonal peaks, notably in the STC/South Tasman zoogeographic region. However, inclusion of surface primary production and chlorophyll-a concentration did not significantly improve GAMs.

Factors co-related to productivity, such as distribution of food sources, breeding and competitive exclusion are likely to contribute to species distributions [21], [102], [103]. Adult lanternfishes are capable of significant swimming [30], [104] that may be involved in orientation or migration to preferred habitat. A blend of active habitat selection and passive hydrodynamic processes such as creation of larval transport barriers or aggregating eddies [105], [106], [107] are likely involved in the maintenance of lanternfish zoogeographic regions.

## Supporting Information

Figure S1 **Area-wise dendrogram of MAXENT logistic scores (Ochiai similarity).** Samples labelled by latitude of geographic pixel and colour-coded for position with respect to water masses and fronts (see text).
(TIF)

Table S1 **Lanternfish species with significant affinity to the four modelled zoogeographic regions and a description of their general distribution.**
(DOCX)
