# Supplementary figures and images for: Lanternfish (Myctophidae) Zoogeography off Eastern Australia: A Comparison with Physicochemical Biogeography
Source: PLoS One. 2013 Dec 11;8(12):e80950. doi: 10.1371/journal.pone.0080950 (PMC3859470; doi:10.1371/journal.pone.0080950)

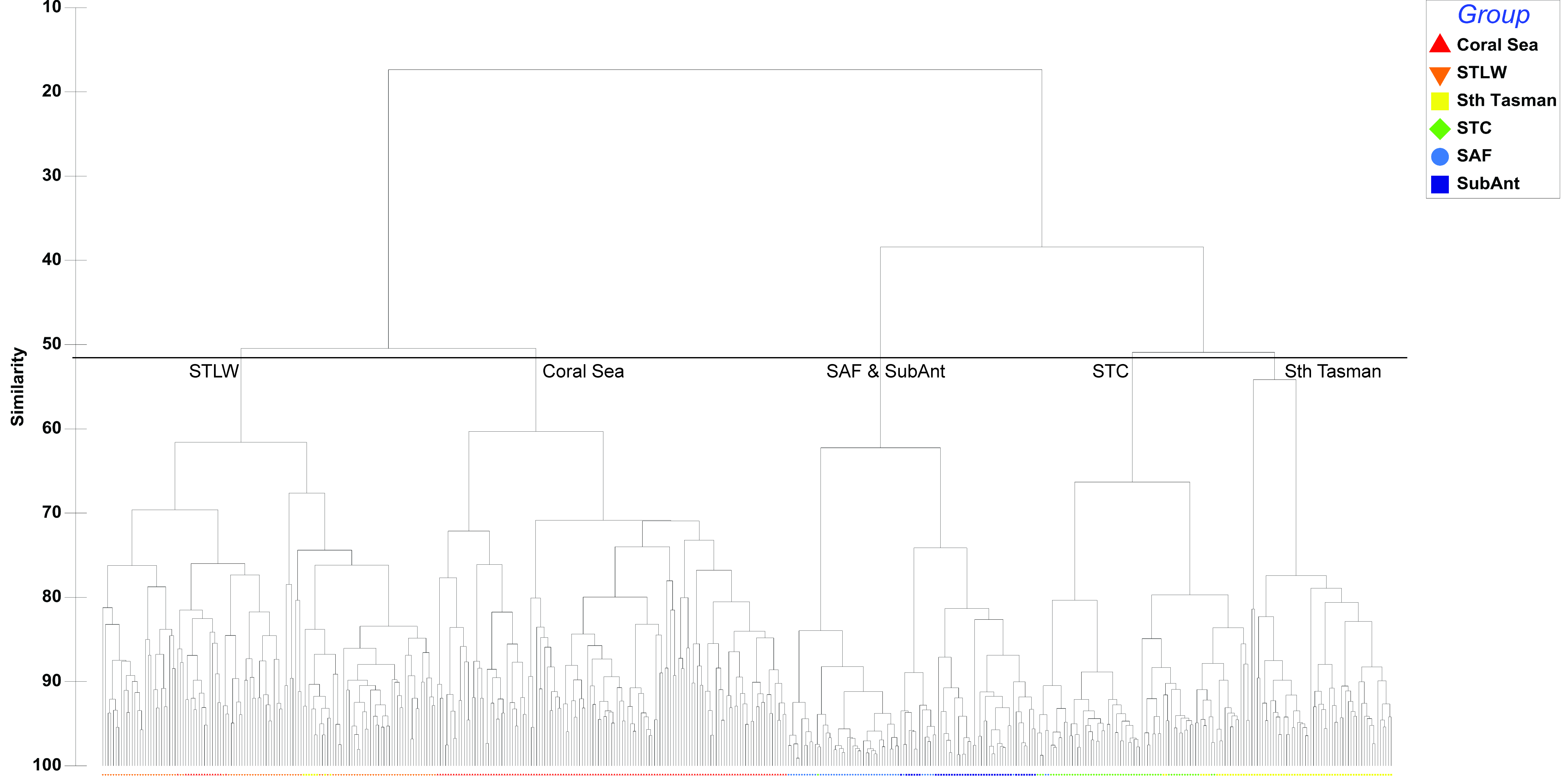

Supplement: Figure S1 — Area-wise dendrogram of MAXENT logistic scores (Ochiai similarity). Samples labelled by latitude of geographic pixel and colour-coded for position with respect to water masses and fronts (see text). (TIF) [file pone.0080950.s001.tif]
