# Supplementary material for: Lanternfish (Myctophidae) Zoogeography off Eastern Australia: A Comparison with Physicochemical Biogeography
Source: PLoS One. 2013 Dec 11;8(12):e80950. doi: 10.1371/journal.pone.0080950 (PMC3859470; doi:10.1371/journal.pone.0080950)
Supplement: Table S1 — Lanternfish species with significant affinity to the four modelled zoogeographic regions and a description of their general distribution. (DOCX) [file pone.0080950.s002.docx]

| **Zoogeographic region** | **Species** | **Distribution** |
| --- | --- | --- |
| Coral Sea Region | *Diaphus aliciae* | Tropical |
|  | *Diaphus jenseni* | Tropical |
|  | *Diaphus malayanus* | Tropical |
|  | *Diaphus regani* | Tropical |
|  | *Diaphus signatus* | Tropical |
|  | *Diogenichthys panurgus* | Tropical |
|  | *Lampanyctus vadulus* | Tropical |
|  | *Myctophum aurolaternatum* | Tropical |
|  | *Myctophum lychnobium* | Tropical |
|  | *Nannobrachium nigrum* | Tropical |
| STLW Region | *Bolinichthys nikolayi* | Subtropical |
|  | *Centrobranchus nigroocellatus* | Subtropical |
|  | *Diaphus anderseni* | Subtropical |
|  | *Diaphus bertelseni* | Subtropical |
|  | *Diaphus brachycephalus* | Subtropical |
|  | *Diaphus fragilis* | Subtropical |
|  | *Diaphus lucidus* | Subtropical |
|  | *Diaphus mollis* | Subtropical |
|  | *Diaphus parri* | Subtropical |
|  | *Diaphus perspicillatus* | Subtropical |
|  | *Diogenichthys atlanticus* | Subtropical |
|  | *Hygophum reinhardtii* | Subtropical |
|  | *Lobianchia gemellarii* | Subtropical |
|  | *Myctophum asperum* | Subtropical |
|  | *Myctophum nitidulum* | Subtropical |
|  | *Myctophum selenops* | Subtropical |
|  | *Triphoturus nigrescens* | Subtropical |
|  | *Diaphus efflugens* | Temperate |
|  | *Diaphus kapalae* | Temperate |
|  | *Diaphus meadi* | Temperate |
|  | *Hygophum hygomii* | Temperate |
|  | *Lampadena notialis* | Temperate |
|  | *Lampanyctus pusillus* | Temperate |
|  | *Myctophum phengodes* | Temperate |
|  | *Notoscopelus caudispinosus* | Temperate |
|  | *Notoscopelus resplendens* | Temperate |
|  | *Scopelopsis multipunctatus* | Temperate |
| STC/South Tasman Region | *Electrona paucirastra* | STC |
|  | *Electrona risso* | STC |
|  | *Hygophum hanseni* | STC |
|  | *Lampanyctodes hectoris* | STC |
|  | *Lampichthys procerus* | STC |
|  | *Lobianchia dofleini* | STC |
|  | *Metelectrona ventralis* | STC |
|  | *Protomyctophum normani* | STC |
|  | *Protomyctophum subparallelum* | STC |
|  | *Symbolophorus barnardi* | STC |
| Subantarctic Region | *Electrona antarctica* | SAF |
|  | *Gymnoscopelus braueri* | SAF |
|  | *Gymnoscopelus fraseri* | SAF |
|  | *Gymnoscopelus nicholsi* | SAF |
|  | *Krefftichthys anderssoni* | SAF |
|  | *Protomyctophum andriashevi* | SAF |
|  | *Protomyctophum bolini* | SAF |
|  | *Protomyctophum gemmatum* | SAF |
|  | *Protomyctophum tenisoni* | SAF |
|  | *Diaphus ostenfeldi* | Sthn Ocean |
|  | *Electrona subaspera* | Sthn Ocean |
|  | *Gymnoscopelus bolini* | Sthn Ocean |
|  | *Gymnoscopelus microlampas* | Sthn Ocean |
|  | *Gymnoscopelus piabilis* | Sthn Ocean |
|  | *Hintonia candens* | Sthn Ocean |
|  | *Lampanyctus intricarius* | Sthn Ocean |
|  | *Lampanyctus macdonaldi* | Sthn Ocean |
|  | *Nannobrachium achirus* | Sthn Ocean |
|  | *Protomyctophum parallelum* | Sthn Ocean |
